# Supplementary material for: Consumption of Breast Milk Is Associated with Decreased Prevalence of Autism in Fragile X Syndrome
Source: Nutrients. 2021 May 24;13(6):1785. doi: 10.3390/nu13061785 (PMC8225095; doi:10.3390/nu13061785)
Supplement: Supplementary file 1 [file nutrients-13-01785-s001.zip › nutrients-1202237-supplementary.pdf]

Supplementary Data

Consumption of Breast Milk is Associated with Decreased Prevalence of Autism in Fragile X Syndrome

Cara J. Westmark [1,2]

[1] Department of Neurology, University of Wisconsin, Madison, WI, 53706 USA; westmark@wisc.edu; Tel.: +1-608-262-9730
[2] Molecular & Environmental Toxicology Center, University of Wisconsin, Madison, WI, 53706 USA

Supplementary Table S1. Analysis of breast milk duration as a function of autism status.

Phenotype	no Autism (*n*=68)	Autism (*n*=64)	*p* [b]
BM [a] < 3 months (%)	16	22	0.40
BM ≥ 3 months (%)	84	78	0.40
BM ≥ 6 months (%)	79	67	0.11
BM ≥ 12 months (%)	56	28	0.0013

[a] BM = breast milk. [b] Chi-squared *p* values.

Supplementary Table S2. Timeframe of seizures in FXS study population as a function of breast milk.

Age of Onset of Seizures	BM [a] % (*n*=17)	No BM % (*n*=8)	*p*	OR	95% CI
0 - 2 weeks (%)	0	0	1.0 [b]	n/a	n/a
0 - 12 months (%)	0	0	1.0 [b]	n/a	n/a
0 - 3 years (%)	18	13	1.0 [b]	1.5	0.13-17.2
Mean age in years (SEM)	6.5 (0.80)	9.1 (2.6)	0.23 [c]	n/a	n/a

[a] BM = breast milk. [b] Fisher exact test. [c] Student t-test.

Supplementary Table S3. Analysis of FXS comorbidities as a function of exclusive milk use.

Phenotype	BM [a] % (*n*)	CM [b] % (*n*)	SM [c] % (N)	*p* [d]
autism	28 (40)	47 (15)	50 (6)	0.31
food allergies	13 (38)	7.7 (13)	40 (5)	0.29
GI problems	23 (40)	50 (16)	17 (6)	0.11
seizures	9.5 (42)	6.3 (16)	33 (6)	0.18
allergies	33 (40)	63 (16)	50 (6)	0.12

[a] BM = breast milk. [b] CM = cow milk formula. [c] SM = soy-based infant formula. [d] Fisher exact test (3 × 2 contingency table) was used.

Supplementary File 1

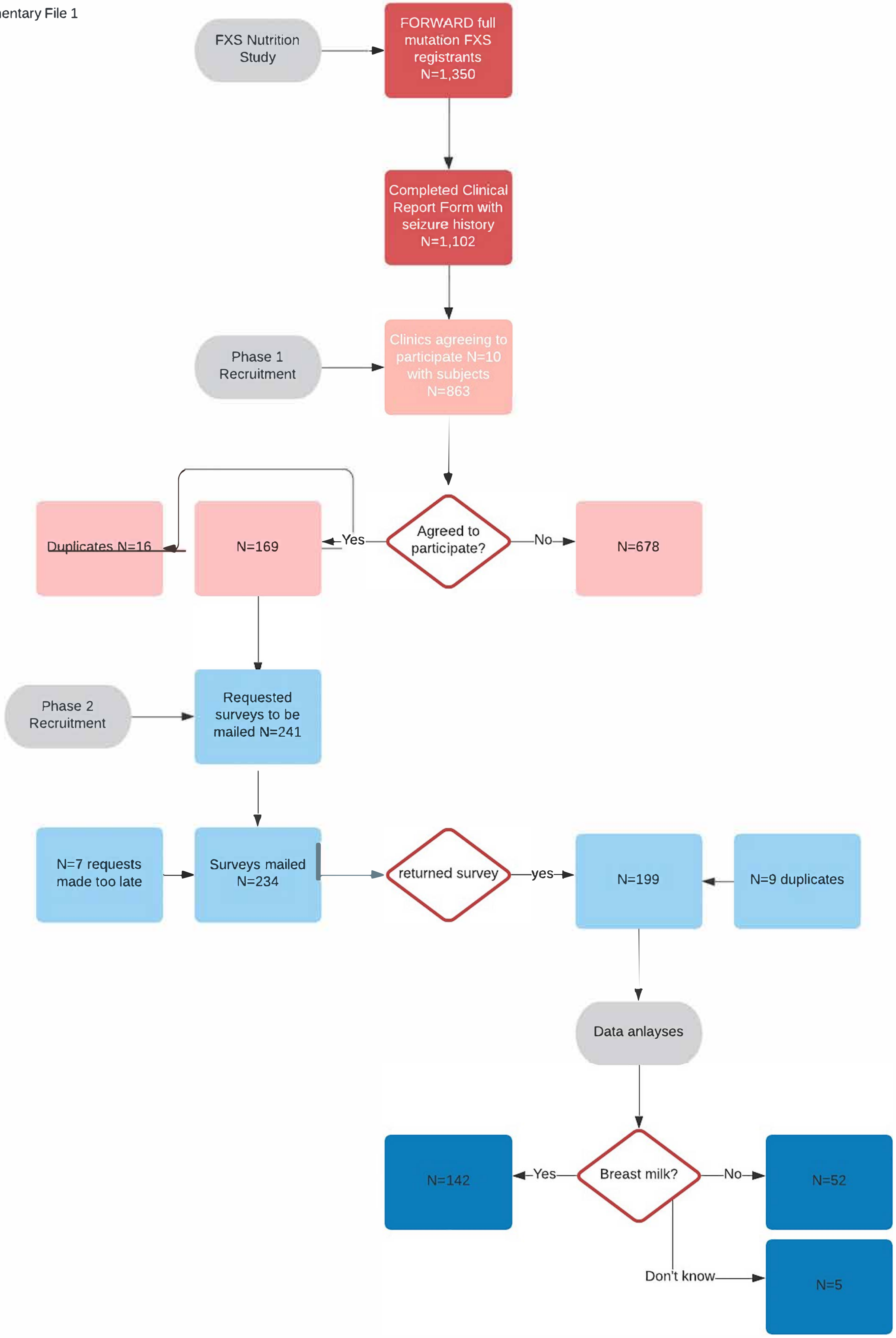

STROBE Statement—checklist of items that should be included in reports of observational studies

	Item No	Recommendation	
**Title and abstract**	1	(*a*) Indicate the study's design with a commonly used term in the title or the abstract	√
		(*b*) Provide in the abstract an informative and balanced summary of what was done and what was found	√
**Introduction**			
Background/rationale	2	Explain the scientific background and rationale for the investigation being reported	√
Objectives	3	State specific objectives, including any prespecified hypotheses	√
**Methods**			
Study design	4	Present key elements of study design early in the paper	√
Setting	5	Describe the setting, locations, and relevant dates, including periods of recruitment, exposure, follow-up, and data collection	√
Participants	6	(*a*) *Cohort study*—Give the eligibility criteria, and the sources and methods of selection of participants. Describe methods of follow-up<br>*Case-control study*—Give the eligibility criteria, and the sources and methods of case ascertainment and control selection. Give the rationale for the choice of cases and controls<br>*Cross-sectional study*—Give the eligibility criteria, and the sources and methods of selection of participants	√
		(*b*) *Cohort study*—For matched studies, give matching criteria and number of exposed and unexposed<br>*Case-control study*—For matched studies, give matching criteria and the number of controls per case	N/A
Variables	7	Clearly define all outcomes, exposures, predictors, potential confounders, and effect modifiers. Give diagnostic criteria, if applicable	√
Data sources/ measurement	8*	For each variable of interest, give sources of data and details of methods of assessment (measurement). Describe comparability of assessment methods if there is more than one group	√
Bias	9	Describe any efforts to address potential sources of bias	√
Study size	10	Explain how the study size was arrived at	Citation 19
Quantitative variables	11	Explain how quantitative variables were handled in the analyses. If applicable, describe which groupings were chosen and why	√
Statistical methods	12	(*a*) Describe all statistical methods, including those used to control for confounding	√
		(*b*) Describe any methods used to examine subgroups and interactions	√
		(*c*) Explain how missing data were addressed	√

(*d*) *Cohort study*—If applicable, explain how loss to follow-up was addressed<br>*Case-control study*—If applicable, explain how matching of cases and controls was addressed<br>*Cross-sectional study*—If applicable, describe analytical methods taking account of sampling strategy	N/A
(*e*) Describe any sensitivity analyses	NA

Continued on next page

**Results**			
Participants	13*	(a) Report numbers of individuals at each stage of study—eg numbers potentially eligible, examined for eligibility, confirmed eligible, included in the study, completing follow-up, and analysed	Supplementary File 1
		(b) Give reasons for non-participation at each stage	Survey question blank or subject chose not participate
		(c) Consider use of a flow diagram	Supplementary File 1
Descriptive data	14*	(a) Give characteristics of study participants (eg demographic, clinical, social) and information on exposures and potential confounders	Citation 19
		(b) Indicate number of participants with missing data for each variable of interest	Tables indicate number of subjects
		(c) *Cohort study*—Summarise follow-up time (eg, average and total amount)	N/A
Outcome data	15*	Cohort study—Report numbers of outcome events or summary measures over time	N/A
		Case-control study—Report numbers in each exposure category, or summary measures of exposure	Tables 1-4, 6, 8-10
		Cross-sectional study—Report numbers of outcome events or summary measures	N/A
Main results	16	(*a*) Give unadjusted estimates and, if applicable, confounder-adjusted estimates and their precision (eg, 95% confidence interval). Make clear which confounders were adjusted for and why they were included	Tables 2-3, 6, 8-9
		(*b*) Report category boundaries when continuous variables were categorized	N/A
		(*c*) If relevant, consider translating estimates of relative risk into absolute risk for a meaningful time period	N/A
Other analyses	17	Report other analyses done—eg analyses of subgroups and interactions, and sensitivity analyses	√
**Discussion**			
Key results	18	Summarise key results with reference to study objectives	√
Limitations	19	Discuss limitations of the study, taking into account sources of potential bias or imprecision. Discuss both direction and magnitude of any potential bias	√
Interpretation	20	Give a cautious overall interpretation of results considering objectives, limitations, multiplicity of analyses, results from similar studies, and other relevant evidence	√
Generalisability	21	Discuss the generalisability (external validity) of the study results	√
**Other information**			
Funding	22	Give the source of funding and the role of the funders for the present study and, if applicable, for the original study on which the present article is based	√

*Give information separately for cases and controls in case-control studies and, if applicable, for exposed and unexposed groups in cohort and cross-sectional studies.

Note: An Explanation and Elaboration article discusses each checklist item and gives methodological background and published examples of transparent reporting. The STROBE checklist is best used in conjunction with this article (freely available on the Web sites of PLoS Medicine at http://www.plosmedicine.org/, Annals of Internal Medicine at http://www.annals.org/, and Epidemiology at http://www.epidem.com/). Information on the STROBE Initiative is available at www.strobe-statement.org.

Fragile X Syndrome Nutrition Study

Please complete the questionnaire below thinking about your child with Fragile X Syndrome.

For consistency, the words “child” and “parent” are used throughout the questionnaire although we recognize that you may be reporting about a person who is now a teen or adult, and that you may be a relative or caretaker, rather than the parent of the child.

1. Does your child with Fragile X Syndrome also have…

	Yes	No	Don’t know
**a.** …Autism?	○	○	○
**b.** …Down Syndrome?	○	○	○
**c.** …Epilepsy?	○	○	○
**d.** …food allergies?	○	○	○
**e.** …Diabetes?	○	○	○

2. Does your child with Fragile X Syndrome also have a history of gastrointestinal problems?

○ Yes
○ No → **Go to question 4**
○ Don’t know → **Go to question 4**

3. How old was your child when these problems started?

[ ] Weeks / Months / Years ***(Please circle one.)***

4. Does your child with Fragile X Syndrome also have a history of seizures?

○ Yes
○ No → **Go to question 15 on page 3**
○ Don’t know → **Go to question 15 on page 3**

5. The next questions ask about the type of seizures your child has had. If you do not recall if you were ever told they had that type of seizure, please check don’t know. Have you been told your child with Fragile X Syndrome has ever had…

	Yes	No	Don’t know
**a.** …febrile seizures (with fever)?	○	○	○
**b.** …atonic seizures (drop attacks)?	○	○	○
**c.** …generalized tonic clonic seizures (grand mal)?	○	○	○
**d.** …absence seizures (petit mal)?	○	○	○
**e.** …simple partial seizures (focal)?	○	○	○
**f.** …complex partials?	○	○	○
**g.** …infantile spasms?	○	○	○

6. **Does your child have a history of some <u>other</u> type of seizure? Please describe their other type of seizure.**

[ ]

7. **At what age did your child have his or her <u>first</u> seizure?**

[ ] Weeks / Months / Years ***(Please circle one.)***

8. **Sometimes children have periods where their seizures are more frequent. Was there a time when your child's seizures were more frequent than they had been previously, or than they have been since that time?**

○ Yes
○ No → **Go to question 11**
○ Don't know → **Go to question 11**

9. **At about what age were your child's seizures happening most often?**

[ ] Weeks / Months / Years ***(Please circle one.)***

10. **Thinking about the time when your child's seizures were happening most often, what was the average number of seizures your child had?**

[ ] Per day/ Per week / Per month / Per year ***(Please circle one.)***

11. **Was your child given medication to treat the seizures?**

○ Yes
○ No → **Go to question 14 on page 3**
○ Don't know → **Go to question 14 on page 3**

12. **Were multiple medications required to treat the seizures?**

○ Yes
○ No
○ Don't know

13. **Did the medication(s) reduce the seizures?**

○ Yes
○ No
○ Don't know

14. In what month, day and year did your child have his or her last seizure?

MM	DD	YYYY

15. Does your child with Fragile X Syndrome also have a history of allergies?

◯ Yes
◯ No → **Go to question 18**
◯ Don't know → **Go to question 18**

16. How old was your child when he or she first began to experience allergies?

[ ] Weeks / Months / Years ***(Please circle one.)***

17. Some examples of allergens might include pollen, dust, pets, latex, eggs, fish, gluten or wheat, milk, nuts, medications, and many others.

What are the allergens your child reacts to now or has in the past?

18. For the next questions, please think back to your child's first year of life.

During the first year of life, was your child with Fragile X Syndrome fed any breastmilk?

◯ Yes
◯ No → **Go to question 23 on page 4**
◯ Don't know → **Go to question 23 on page 4**

19. How old was your child when he or she began to be fed breastmilk?

[ ] Days/ Weeks / Months ***(Please circle one.)***

20. Some examples of why people might choose to feed their child breastmilk include doctor recommendations, family recommendations, or a belief that it is healthiest for the baby.

Why was your child fed breastmilk?

21. How old was your child when he or she stopped being fed breastmilk?

[ ] Days/ Weeks / Months ***(Please circle one.)***

22. Some examples of why people might choose to stop feeding their child breastmilk include being painful for the mother, or the baby not getting enough milk.

What was the reason your child stopped being fed breastmilk?

[ ]

23. Was your child with Fragile X Syndrome fed any cow milk formula in his or her first year of life?

- ○ Yes
- ○ No → **Go to question 28 on page 5**
- ○ Don't know → **Go to question 28 on page 5**

24. How old was your child when he or she began to be fed cow milk formula?

[ ] Days/ Weeks ***(Please circle one.)***

25. Some examples of why people might choose to feed their child cow milk formula include doctor recommendations, family recommendations, or an inability to breastfeed.

Why was your child fed cow milk formula?

[ ]

26. How old was your child when he or she stopped being fed cow milk formula?

[ ] Days/ Weeks / Months ***(Please circle one.)***

27. Some examples of why people might choose to stop feeding their child cow milk formula include allergy, intolerance, constipation, diarrhea, too much mucus, gas, too much spit up, vomiting, or parental choice.

What was the reason your child stopped being fed cow milk formula?

[ ]

28. Was your child with Fragile X Syndrome fed any soy-based formula in his or her first year of life?

- ○ Yes
- ○ No → **Go to question 33**
- ○ Don't know → **Go to question 33**

29. How old was your child when he or she began to be fed soy-based formula?

[ ] Days/ Weeks ***(Please circle one.)***

30. Some examples of why people might choose to feed their child soy-based formula include problems with other foods such as allergy, intolerance, constipation, diarrhea, too much mucus, gas, too much spit up, vomiting, or parental choice.

Why was your child fed soy-based formula?

[ ]

31. How old was your child when he or she stopped being fed soy-based formula?

[ ] Days/ Weeks / Months ***(Please circle one.)***

32. Some examples of why people might choose to stop feeding their child soy-based formula include allergy, intolerance, constipation, diarrhea, too much mucus, gas, too much spit up, vomiting, or parental choice.

What was the reason your child stopped being fed soy-based formula?

[ ]

33. Some examples of specialty formulas include amino acid, rice, or meat-based formulas. Was your child with Fragile X Syndrome fed any specialty formulas in his or her first year of life?

- ○ Yes
- ○ No → **Go to question 38 on page 6**
- ○ Don't know → **Go to question 38 on page 6**

34. How old was your child when he or she began to be fed a specialty formula?

[ ] Days/ Weeks ***(Please circle one.)***

35. Some examples of why people might choose to feed their child specialty formulas include problems with other foods such as allergy, intolerance, constipation, diarrhea, too much mucus, gas, too much spit up, vomiting, or parental choice.

Why was your child fed other specialty formulas?

36. How old was your child when he or she stopped being fed a specialty formula?

Days/ Weeks / Months ***(Please circle one.)***

37. Some examples of why people might choose to stop feeding their child other specialty formulas include allergy, intolerance, constipation, diarrhea, too much mucus, gas, too much spit up, vomiting, or parental choice.

What was the reason your child stopped being fed a specialty formula?

38. For the next questions, please think about the biological family of your child with Fragile X Syndrome.

Did anyone in the child's biological family including mother, father, brother, or sister have any of the following, and if yes, what was the relationship of that family member to the child?

Condition	Don't know	No	Yes		Specify Relationship to Child
Asthma	○	○	○	→	
Allergies	○	○	○	→	
Thyroid Condition	○	○	○	→	
Seizures	○	○	○	→	
Autoimmune Condition	○	○	○	→	
Juvenile onset Diabetes	○	○	○	→	
Adult onset Diabetes	○	○	○	→	

39. If you indicated a family member had an autoimmune condition, what was it?

40. For the next questions, we want you to try to remember how your child behaved when he or she was about 3 years old. If you were not with your child at that age, or you do not remember how they behaved, you may skip any one of these questions or skip this entire section and go on to question 47 on page 9.

About how <u>often</u> did your child…

	Never	Rarely	Sometimes	Very often	Extremely often
a. …talk?	○	○	○	○	○
b. …say his or her name when asked?	○	○	○	○	○
c. …respond when spoken to, for example, did your child look at you when you called his or her name?	○	○	○	○	○
d. …speak in sentences of at least 3 words?	○	○	○	○	○
e. …use words to request things, for example when he or she wanted a cookie?	○	○	○	○	○

41. Still remembering when your child with Fragile X Syndrome was 3 years old, about how <u>often</u> did your child…

	Never	Rarely	Sometimes	Very often	Extremely often
a. …correctly identify people or objects when you pointed to them and asked what they were, for example, "mommy", "daddy", "dog", or "airplane"?	○	○	○	○	○
b. …follow simple directions, for example "sit down" or "get your shoes"?	○	○	○	○	○
c. …point at things to request them, for example, a toy on a shelf?	○	○	○	○	○
d. …copy others, for example, clapping their hands or waving?	○	○	○	○	○
e. …play pretend, for example, rocking a doll to sleep or feeding a stuffed animal?	○	○	○	○	○
f. …have savant ability, a restricted skill superior to their age group, for example reading early, or memorizing books?	○	○	○	○	○

42. If you indicated your child with Fragile X Syndrome has or had a savant ability, what is it?

43. Still remembering when your child with Fragile X Syndrome was 3 years old, how <u>often</u> did your child…

	Never	Rarely	Sometimes	Very often	Extremely often
a. …like motion activities, for example, to be swung or bounced?	○	○	○	○	○
b. …walk?	○	○	○	○	○
c. …toe walk?	○	○	○	○	○
d. …pick up small objects, for example, Cheerios?	○	○	○	○	○
e. …feed him or herself with a spoon?	○	○	○	○	○
f. …help dress him or herself, for example pull up their pants?	○	○	○	○	○

44. Still remembering when your child with Fragile X Syndrome was 3 years old, how <u>often</u> did your child…

	Never	Rarely	Sometimes	Very often	Extremely often
a. …get upset by loud noises, for example, the vacuum cleaner or microwave?	○	○	○	○	○
b. …do rocking, hand flapping or spinning over and over again?	○	○	○	○	○
c. …cry excessively over small hurts?	○	○	○	○	○
d. …have temper outbursts if he or she did not get their way?	○	○	○	○	○
e. …isolate him or herself?	○	○	○	○	○
f. …try to injure him or herself, for example, head banging?	○	○	○	○	○

45. Still remembering when your child with Fragile X Syndrome was 3 years old, how <u>often</u> did your child…

	Never	Rarely	Sometimes	Very often	Extremely often
a. …get upset by minor changes to their daily routine?	○	○	○	○	○
b. …have difficulty expressing his or her needs and desires?	○	○	○	○	○
c. …hate crowds, for example, difficulties in restaurants or the grocery store?	○	○	○	○	○
d. …not like to be touched or held?	○	○	○	○	○
e. …like to play with other children?	○	○	○	○	○

46. Still remembering back to when your child with Fragile X Syndrome was 3 years old, how often did you think your child…

	Never	Rarely	Sometimes	Very often	Extremely often
a. …had an anxiety problem?	◯	◯	◯	◯	◯
b. …had a hearing problem?	◯	◯	◯	◯	◯
c. …had a vision problem?	◯	◯	◯	◯	◯
d. …had a learning problem?	◯	◯	◯	◯	◯

47. What is your relationship to the child with Fragile X Syndrome?

◯ Mother
◯ Father
◯ Sister
◯ Brother
◯ Grandparent
◯ Caretaker
◯ Teacher
◯ Other relationship → Please tell us: ______

48. What is the sex of your child with Fragile X Syndrome?

◯ Female
◯ Male

49. Which of the following describe your child's race or ethnicity? Please check all that apply

☐ American Indian or Alaskan Native
☐ Asian
☐ Black or African American
☐ Hispanic or Latino
☐ Native Hawaiian or Other Pacific Islander
☐ White
☐ Other race or ethnicity: Please tell us: → ______

50. What is your child's date of birth?

MM DD YYYY

51. What was your child's length in inches at birth? If you are not sure, please provide your best estimate.

______ Inches

52. What was your child's weight at birth? If you are not sure, please provide your best estimate.

[ ] Pounds [ ] Ounces

53. What is your child's current height? If you are not sure, please provide your best estimate.

[ ] Feet [ ] Inches

54. What is your child's current weight? If you are not sure, please provide your best estimate.

[ ] Pounds

55. What is the date on which you completed this questionnaire?

[ ] [ ] [ ]
MM DD YYYY

Thank you for agreeing to participate in our research study on Fragile X Syndrome.
We want you to know that your time and effort are greatly appreciated.
